# Validation of a Dynamic Planning Navigation Strategy Applied to Mobile Terrestrial Robots

**DOI:** 10.3390/s18124322

**Published:** 2018-12-07

**Authors:** Caroline A. D. Silva, Átila V. F. M. de Oliveira, Marcelo A. C. Fernandes

**Affiliations:** Department of Computer Engineering and Automation, Center of Technology, Federal University of Rio Grande do Norte—UFRN, 59078-970, Brazil; carolads@gmail.com (C.A.D.S.); avfmo.engcomp@gmail.com (Á.V.F.M.d.O.)

**Keywords:** genetic algorithms, mobile robots, autonomous navigation, dynamic planning

## Abstract

This work describes the performance of a DPNA-GA (Dynamic Planning Navigation Algorithm optimized with Genetic Algorithm) algorithm applied to autonomous navigation in unknown static and dynamic terrestrial environments. The main aim was to validate the functionality and robustness of the DPNA-GA, with variations of genetic parameters including the crossover rate and population size. To this end, simulations were performed of static and dynamic environments, applying the different conditions. The simulation results showed satisfactory efficiency and robustness of the DPNA-GA technique, validating it for real applications involving mobile terrestrial robots.

## 1. Introduction

In most of the studies concerning Genetic Algorithms (GAs) encountered in the literature, global or local planning strategies are employed. The former provides optimum routes, at high computational cost associated with a priori knowledge of the environment, while the latter provides suboptimal routes, at lower computational cost and with complete, or almost complete, lack of knowledge concerning the environment [1,2]. Global or local planning can be applied to static and dynamic environments, although in the case of dynamic environments, global planning strategies require the use of external observation devices to periodically transmit the current state of the environment to the robot [3].

Several studies [1,3,4,5,6,7,8] have described navigation strategies employing GAs, with global planning in which the individuals (or chromosomes) are composed of all the possible routes between the initial and final points. In all cases, a priori knowledge is required of the environment, which is represented using a bidimensional grid. Several of the proposed techniques are specific to static environments [1,4,5,6,7,9], while the proposal presented in Refs. [3,8] is aimed at dynamic environments, although an external observation device is needed to transmit the state of the environment to the robot at a speed faster than the speed of changes in the environment. Although efficient results have been reported in these earlier studies, three issues need to be highlighted. The first is that the size of the individual is variable and is a function of the length of the route (the greater the complexity of the environment, the greater the length) and the resolution of the grid associated with the displacement of the robot. This can significantly increase the spatial and temporal complexity of the GA, making it unviable for use in limited hardware systems such as microcontrollers (MCUs), digital signal processors (DSPs), and others. The second issue is that it is not always possible to obtain the a priori knowledge of the environment that is important for global planning strategies. The third point is that these global strategies are better suited to static environments, due to the necessity of using external observation equipment for dynamic environments.

Navigation strategies with GAs based on local planning have been presented for dynamic [10,11] and static [2,12] environments. In these proposals, the individuals possess a dynamic size (dynamic dimension) and store the nodes that compose the route. Despite making use of local planning strategies, these proposals have the same problem described for the global planning methods, where the complexity of the GA is a function of the complexity of the environment and the resolution of the displacement of the robot. Another strategy is shown in Ref. [13] where the chromosomes are formed with the obstacle distances (left, right, front), angle and direction. This approach uses the individuals (chromosomes) with a static dimension which enables have a computation complexity less than the works proposed in Refs. [2,10,11,12].

The works presented in Refs. [14,15] propose global navigation planning, similar to the works [1,4,5,6,7,9] with other population-based metaheuristics algorithms [16]. The research [14] uses the ant colony optimization, and the work [15] uses the pseudo-bacterial genetic algorithm. Already, the work presented in Ref. [17] proposes the local planning for mobile robot navigation using firefly algorithm. Other approaches using artificial intelligence techniques such as machine learning, fuzzy systems, Q-learning are presented in Refs. [18,19,20,21].

Different to the studies cited above, the work described in Ref. [22] presents a navigation strategy called the Dynamic Planning Navigation Algorithm optimized with Genetic Algorithm (DPNA-GA). This strategy employs a navigation scheme with local planning, in which the environment is a priori unknown, and the sizes of the individuals are fixed (only representing possible local objectives through which the robot could move) and are independent of the complexity of the environment or the size of the route. Another important point is that the DPNA-GA can be applied to static or dynamic environments, given that the planning is reformulated at each displacement. Even though navigation techniques with local planning provide suboptimal solutions, it can be seen from the results presented that in many cases it is possible to obtain routes very close to the optimum.

Nonetheless, the work presented in Ref. [22] does not provide details of the operation of the DPNA-GA as a function of the genetic parameters. Therefore, the purpose of this work is to present results obtained using the DPNA-GA for static and dynamic environments, varying the genetic parameters to validate and generalize the technique proposed in Ref. [22].

## 2. DPNA-GA Strategy

To facilitate understanding of the results, this section details the DPNA-GA strategy presented in Refs. [22,23].

It is assumed that the robot possesses a location sensor, which returns its spatial position, $p^{R}=\left(x^{R},y^{R}\right)$, and a set of *n* evenly distributed distance sensors. The navigation strategy based on the DPNA-GA generates a route composed of *M* local displacement events to reach the final objective, $p^{of}=\left(x^{of},y^{of}\right)$. In each *m*-th displacement event, there is a local objective, $p^{ol}\left(m\right)=\left(x^{ol}\left(m\right),y^{ol}\left(m\right)\right)$, to which the robot moves.

The selection of the local objective, $p^{ol}\left(m\right)$, in each *m*-th event, is performed by a GA that considers the current position of the robot, $p^{R}\left(m\right)$, the distance to the final objective, $p^{of}$, and the obstacles detected by the *n* distance sensors. All the positions, $p^{R}\left(m\right)$, already visited by the robot up to the *m*-th displacement are stored in the vector, $p^{R}$, expressed by

(1) $$\begin{array}{ccc} p^{R} & = & \left[\begin{array}{c} p^{R}\left(0\right)\\ p^{R}\left(1\right)\\ \vdots \\ p^{R}\left(m-1\right)\\ p^{R}\left(m\right) \end{array}\right]\\ & = & \left[\begin{array}{c} \left(x^{R}\left(0\right),y^{R}\left(0\right)\right)\\ \left(x^{R}\left(1\right),y^{R}\left(1\right)\right)\\ \vdots \\ \left(x^{R}\left(m-1\right),y^{R}\left(m-1\right)\right)\\ \left(x^{R}\left(m\right),y^{R}\left(m\right)\right) \end{array}\right] \end{array}$$

and are also used to optimize the GA, avoiding searches in areas that have already been explored.

The algorithm ends when the current position of the robot is the same as the final objective, so that, $p^{R}\left(m\right)=\left(p^{of}\pm \epsilon \right)$ where ϵ is a tolerance factor, or when the number of displacement events exceeds a maximum value, $M_{max}$. The DPNA-GA can be used in both static and dynamic environments, because at each *m*-th displacement event there is a new search for obstacles and for a new local objective, $p^{ol}\left(m\right)$. The steps processed by the DPNA-GA are presented in Algorithm 1 and are described in detail in the following sections.

**Algorithm 1** DPNA-GA 1: m=0 2: $p^{R}=\left[p^{R}\left(0\right)\right]$ 3: **while**
$p^{R}\left(m\right)\neq \left(p^{of}\pm \epsilon \right)\mathrm{AND}\;m<M_{max}$
**do** 4: $p^{DP}\left(m\right)=\mathrm{Scanning}$ 5: $p^{O}\left(m\right)=\mathrm{ObstaclesDetection}\left(p^{DP}\left(m\right)\right)$ 6: $p^{ol}\left(m\right)=\mathrm{LocalObjectiveSearch}\left(p^{O}\left(m\right),p^{DP}\left(m\right),p^{R}\right)$ 7: $p^{R}\left(m+1\right)=\mathrm{Displacement}\left(p^{ol}\left(m\right)\right)$ 8: $p^{R}={\left[p^{R},p^{R}\left(m+1\right)\right]}^{T}$ 9: m=m+1 10: **end while**

### 2.1. Scanning Step

In this step (line 4 of Algorithm 1), the DPNA-GA forces the robot to perform a $360^{\circ }$ scan of the environment around its axis. From this scan, each *j*-th sensor, in the *m*-th event, returns a signal, $s_{j}\left(m\right)$, proportional to the range, $d_{max}$, of the sensor, so that

(2) $$s_{j}\left(m\right)=\left\{\begin{array}{c} d_{j}\left(m\right)\;\mathrm{for}\;d_{j}\left(m\right)\leq d_{max}\\ d_{max}\;\mathrm{for}\;d_{j}\left(m\right)>d_{max} \end{array}\right.$$

where $d_{j}$ is the distance measured by the *j*-th sensor coupled to the robot.

**Figure 1:**
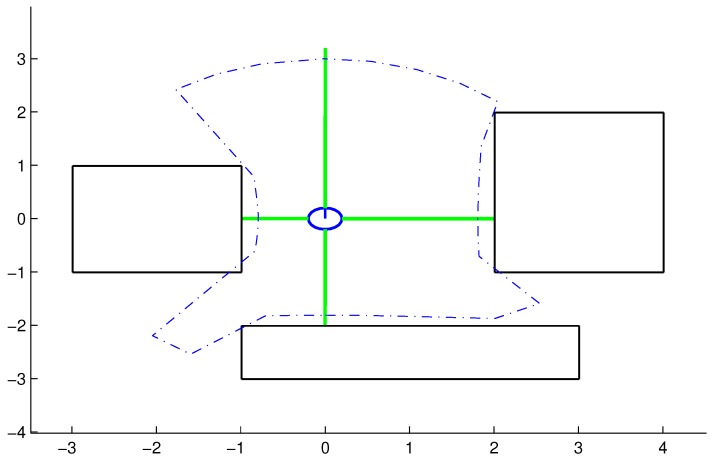
Example of the delimiting polygon (dashed blue line) for the case where n=4 and $\alpha =10^{\circ }$ (p=9).

During the scan, the angular displacement, α, can be expressed by

(3) $$\alpha =\frac{360^{\circ }}{n\cdot p},$$

where *n* is the number of distance sensors and p−1 represents the number of angular displacements that the robot can make on its axis, with the aim of decreasing the resolution and hence requiring a small number of sensors. At the end of the scan process, the DPNA-GA generates a polygon, called the delimiting polygon (DP), composed of a set of *K* points, expressed by the vector

(4) $$\begin{array}{ccc} p^{DP}\left(m\right) & = & \left[\begin{array}{c} p_{0}^{DP}\left(m\right)\\ \vdots \\ p_{k}^{DP}\left(m\right)\\ \vdots \\ p_{K-1}^{DP}\left(m\right) \end{array}\right]\\ & = & \left[\begin{array}{c} \left(x_{0}^{DP}\left(m\right),y_{0}^{DP}\left(m\right)\right)\\ \vdots \\ \left(x_{k}^{DP}\left(m\right),y_{k}^{DP}\left(m\right)\right)\\ \vdots \\ \left(x_{K-1}^{DP}\left(m\right),y_{K-1}^{DP}\left(m\right)\right) \end{array}\right] \end{array}$$

where K=p×n and $\left(x_{k},y_{k}\right)$ represents the in-plane coordinates of the *k*-th point associated with the DP. This polygon is used to delimit the search space associated with the genetic algorithm, such that the points (individuals) generated within the polygon are more suitable than points generated outside it. Meanwhile, it is not only the fact of being within or outside the polygon that defines the suitability of each individual. Also considered are the distances between the point generated and the obstacles detected in the scan, among other factors. Figure 1 illustrates the polygon generated by the DPNA-GA for the case of n=4 and $\alpha =10^{\circ }$.

### 2.2. Detection of Obstacles

The scan step is followed by initiation of the step for detection of the obstacles (line 5 of Algorithm 1). In addition to the DP, a virtual polygon (VP) is generated that describes a circumference centered on the position of the robot ($p^{R}$), with radius $r_{PV}$, slightly less than the range of the sensors, $d_{max}$, such that

(5) $$r_{PV}=d_{max}\left(1-\eta \right),$$

where η is a factor limited to the range 0<η≤0,1. The objective of the VP is to detect only those points of the DP that are associated with obstacles, here denoted $p^{O}$. Hence, after this step, a new set of *L* points is generated, represented by the vector

(6) $$\begin{array}{ccc} p^{O}\left(m\right) & = & \left[\begin{array}{c} p_{0}^{O}\left(m\right)\\ \vdots \\ p_{l}^{O}\left(m\right)\\ \vdots \\ p_{L-1}^{O}\left(m\right) \end{array}\right]\\ & = & \left[\begin{array}{c} \left(x_{0}^{O}\left(m\right),y_{0}^{O}\left(m\right)\right)\\ \vdots \\ \left(x_{l}^{O}\left(m\right),y_{l}^{O}\left(m\right)\right)\\ \vdots \\ \left(x_{L-1}^{O}\left(m\right),y_{L-1}^{O}\left(m\right)\right) \end{array}\right] \end{array}$$

where

(7) $$p_{l}^{O}\left(m\right)=p_{k}^{DP}\left(m\right)\;\mathrm{if}\;f_{ed}\left(p^{R}\left(m\right),p_{k}^{DP}\left(m\right)\right)\leq r_{PV}$$

and L≤K. The function $f_{ed}\left(\cdot ,\cdot \right)$ calculates the Euclidean distance between any two points, which can be expressed by

(8) $$f_{ed}\left(p_{i},p_{b}\right)=\sqrt{{\left(x_{i}-x_{b}\right)}^{2}+{\left(y_{i}-y_{b}\right)}^{2}}$$

**Figure 2:**
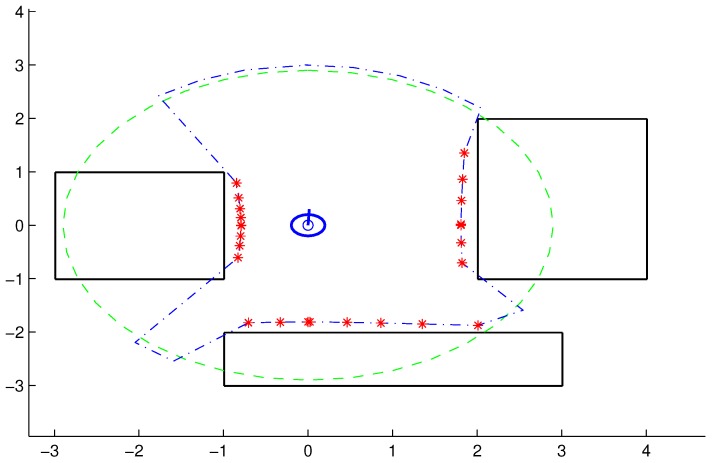
Example illustrating the VP (green line) for a case where η=0.01, and the set of points, $p^{O}$, detected (red asterisks) that avoid the obstacles.

Figure 2 provides an example showing the VP (dashed green circle) and the set of points $p^{O}$ (red asterisks).

### 2.3. Local Objective Search

In this step (line 6 of Algorithm 1), the proposed navigation strategy employs a GA to find a possible local objective, $p^{ol}$, to which the robot will move. For each *m*-th displacement event, the GA is executed with a new population for *H* generations. The individuals are characterized by the vector

(9) $$\begin{array}{ccc} p^{GA}\left(h,m\right) & = & \left[\begin{array}{c} p_{0}^{GA}\left(h,m\right)\\ \vdots \\ p_{j}^{GA}\left(h,m\right)\\ \vdots \\ p_{J-1}^{GA}\left(h,m\right) \end{array}\right]\\ & = & \left[\begin{array}{c} \left(x_{0}^{GA}\left(h,m\right),y_{0}^{GA}\left(h,m\right)\right)\\ \vdots \\ \left(x_{j}^{GA}\left(h,m\right),y_{j}^{GA}\left(h,m\right)\right)\\ \vdots \\ \left(x_{J-1}^{GA}\left(h,m\right),y_{J-1}^{GA}\left(h,m\right)\right) \end{array}\right], \end{array}$$

where $p_{j}^{GA}\left(h,m\right)$ represents the *j*-th individual of the population of size *J*, associated with the *h*-th generation of the *m*-th displacement of the robot. In each generation, *h*, all the individuals are generated according to the nonlinear restriction expressed by

(10) $$r_{d}\geq \sqrt{dx_{j}^{GA}\left(h,m\right)+dy_{j}^{GA}\left(h,m\right)}$$

where

(11) $$dx_{j}^{GA}\left(h,m\right)={\left(x_{j}^{GA}\left(h,m\right)-x^{R}\left(m\right)\right)}^{2}$$

and

(12) $$dy_{j}^{GA}\left(h,m\right)={\left(y_{j}^{GA}\left(h,m\right)-y^{R}\left(m\right)\right)}^{2}$$

This restriction limits the individuals of the population to a circumference with radius $r_{d}$, centered on the position of the robot at the *m*-th instant, $p^{R}\left(m\right)$. Usually, DP occupies most of the circle with radius $r_{d}$, so that a few individuals are created outside DP. Thus, using the coordinates of DP as constraints on population creation would result in a much more complex creation routine, with few practical compensations.

The evaluation function associated with the *j*-th individual of the *h*-th generation in the *m*-th displacement is expressed by

(13) $$\begin{array}{ccc} \;g_{j}\left(h,m\right) & = & d_{j}^{of}\left(h,m\right)+\beta \left(m\right)\frac{1}{d_{j}^{o}\left(h,m\right)}\\ & & +\beta \left(m\right)C_{j}\left(h,m\right)+A_{j}\left(h,m\right) \end{array}$$

where $d_{j}^{of}\left(h,m\right)$ is the Euclidean distance between the *j*-th individual of the *h*-th generation and the final objective, $p^{of}$, such that

(14) $$d_{j}^{of}\left(h,m\right)=f_{ed}\left(p_{j}^{GA}\left(h,m\right),p^{of}\right)$$

and $d_{j}^{o}\left(h,m\right)$ is the shortest Euclidean distance between the *j*-th individual of the *h*-th generation and all the *L* obstacles encountered, which can be expressed as

(15) $$\begin{array}{ccc} d_{j}^{o}\left(h,m\right) & = & \min f_{ed}\left(p_{j}^{GA}\left(h,m\right),p_{l}^{O}\left(m\right)\right)\\ & & \;\mathrm{for}\;l=0,\ldots ,L-1 \end{array}$$

The variables β(m), $C_{j}\left(h,m\right)$ and $A_{j}\left(h,m\right)$ can be considered as penalty factors added to each *j*-th individual of the GA. If no obstacle is encountered in the *m*-th displacement event (L=0), it is assumed that the optimum evaluation function is simply $d_{j}^{of}\left(h,m\right)$, such that

(16) $$\beta \left(m\right)=\left\{\begin{array}{c} 1\;\mathrm{for}\;L\neq 0\\ 0\;\mathrm{for}\;L=0 \end{array}\right.$$

Starting from the principle that the circumferences with radius $r_{d}$, centered in the vector of the center, $p^{R}$, are areas that have already been visited, the penalty vector, $C_{j}\left(h,m\right)$, can be characterized as follows

(17) $$C_{j}\left(h,m\right)=\left\{\begin{array}{cc} 1 & \mathrm{if}\\ & ∄i\in \{0,\cdots ,m-1\}:\\ & f_{ed}\left(p_{j}^{GA}\left(h,m\right),p^{R}\left(i\right)\right)<r_{d}\\ Z & \mathrm{if}\\ & \exists i\in \{0,\cdots ,m-1\}:\\ & f_{ed}\left(p_{j}^{GA}\left(h,m\right),p^{R}\left(i\right)\right)<r_{d} \end{array}\right.$$

where *Z* is a relatively large number. Hence, if an individual, $p_{j}^{GA}\left(h,m\right)$, is located within any of the *m* circumferences of radius $r_{d}$, centered in the vector of the center, $p^{R}$, it will be positively penalized, reducing its chances of selection. Finally, the penalty, $A_{j}\left(h,m\right)$, is referenced to the individuals, $p_{j}^{GA}\left(h,m\right)$, located outside the DP, where

(18) $$A_{j}\left(h,m\right)=\left\{\begin{array}{cc} 0 & \mathrm{if}\;\in DP\\ \infty & \mathrm{if}\;\notin DP \end{array}\right.$$

**Figure 3:**
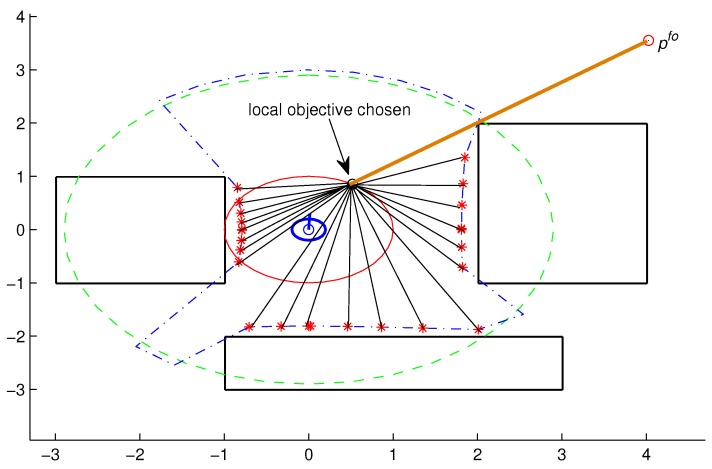
Example illustrating the calculation of the evaluation function for a *j*-th individual, $p_{j}^{GA}\left(h,m\right)$, in relation to the final objective, $p^{of}$, and the obstacles, $p^{O}$.

In this last case, individuals that receive this penalty will have little chance of surviving to the next generation. Figure 3 illustrates the calculation of the evaluation function for a *j*-th individual, $p_{j}^{GA}\left(h,m\right)$.

The evaluation function, presented in Equation (13), follows the same principle as the potential fields technique [24], in which $d_{j}^{of}\left(n,m\right)$ (the Euclidean distance between the *j*-th individual and the final objective, $p^{of}$) represents a traction force to the final point, and $\frac{1}{d_{j}^{o}\left(n,m\right)}$ (the smallest Euclidean distance between the *j*-th individual and all the points associated with the obstacles) represents the greatest force of repulsion between the *j*-th individual and all the obstacles encountered. At the end of *H* generations, the point with the smallest evaluation function is selected as the local objective, $p^{ol}\left(m\right)$, associated with the *m*-th displacement event.

### 2.4. Displacement

**Figure 4:**
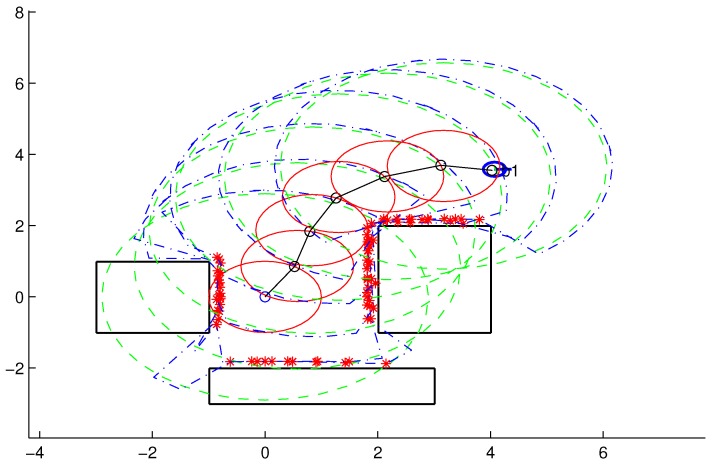
Example illustrating the displacements (M=6) made by the robot towards the final point, $p^{of}$.

The displacement step (line 7 of Algorithm 1) involves movement of the robot to the local objective found in the previous step. After the movement, a new center point, $p^{R}\left(m+1\right)$, is generated, expressed by

(19) $$p^{R}\left(m+1\right)\neq \left(p^{ol}\left(m\right)\pm \epsilon \right)$$

where ϵ is an allowed tolerance in relation to the local objective. This tolerance is essential to the robot with restricted movements, such as nonholonomic robots [24] and errors from real measures. Figure 4 shows a sequence of M=6 displacements to the final point, $p^{of}$.

## 3. Simulation Results

**Figure 5:**
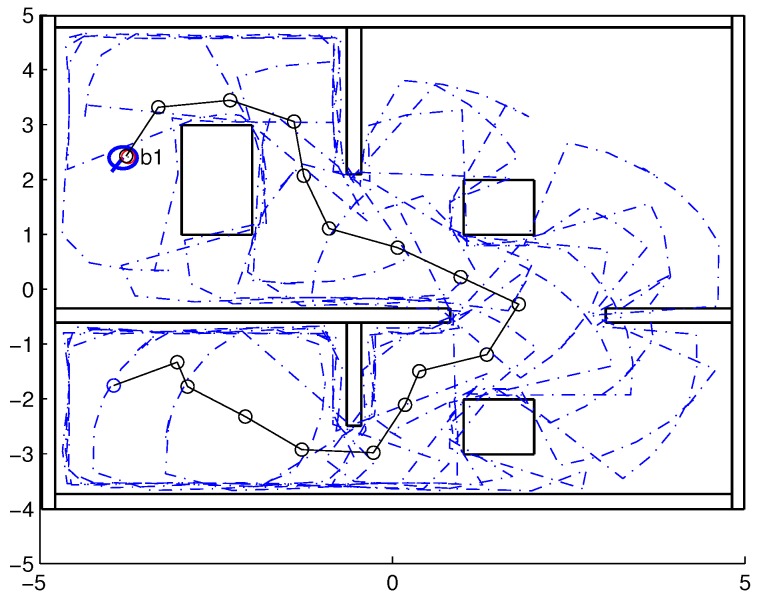
Displacement of the robot in simulation $S_{1}$ (J=30, H=30 and $R_{c}=80\%$). The red circumference—labeled as b1—indicates the final point of the displacement.

**Figure 6:**
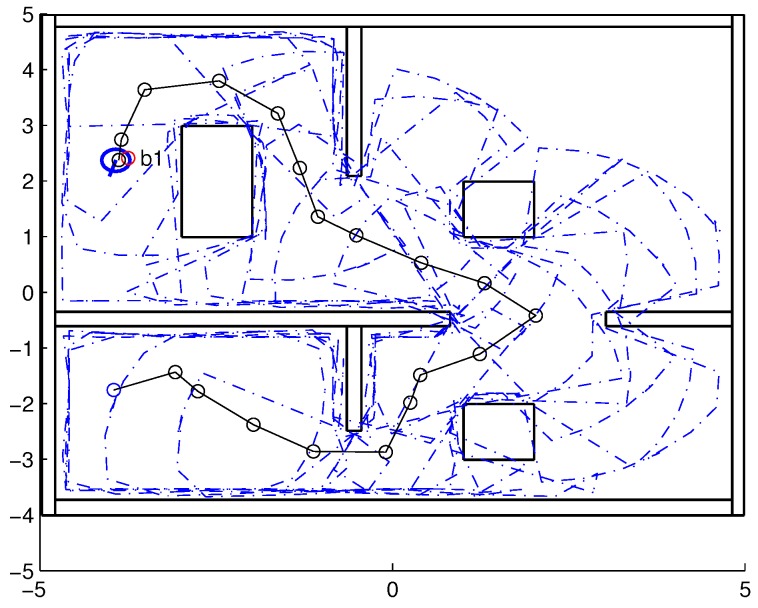
Displacement of the robot in simulation $S_{2}$ (J=10, H=30 and $R_{c}=80\%$). The red circumference—labeled as b1—indicates the final point of the displacement.

**Figure 7:**
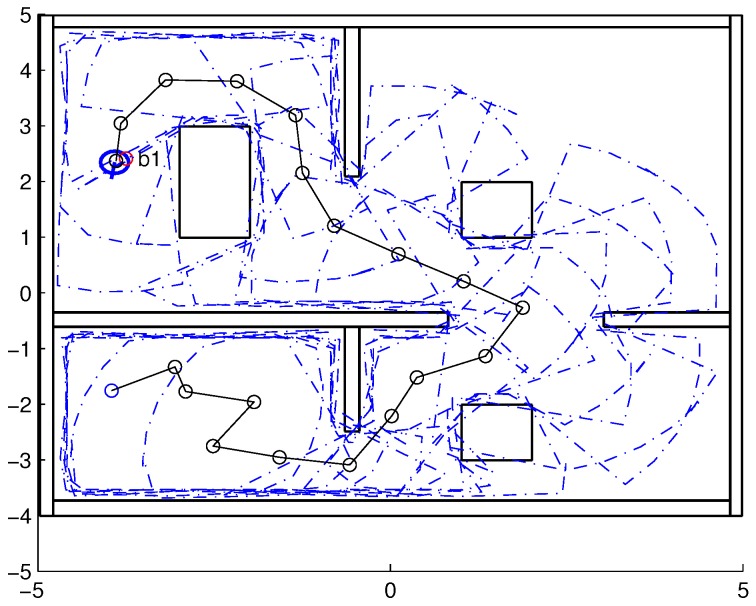
Displacement of the robot in simulation $S_{3}$ (J=30, H=30 and $R_{c}=60\%$). The red circumference—labeled as b1—indicates the final point of the displacement.

**Figure 8:**
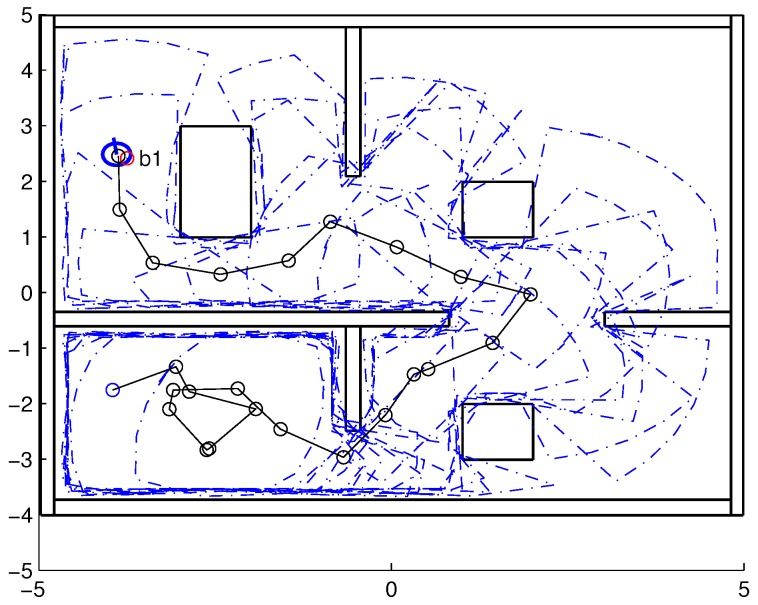
Displacement of the robot in simulation $S_{4}$ (J=10, H=30 and $R_{c}=60\%$). The red circumference—labeled as b1—indicates the final point of the displacement.

**Figure 9:**
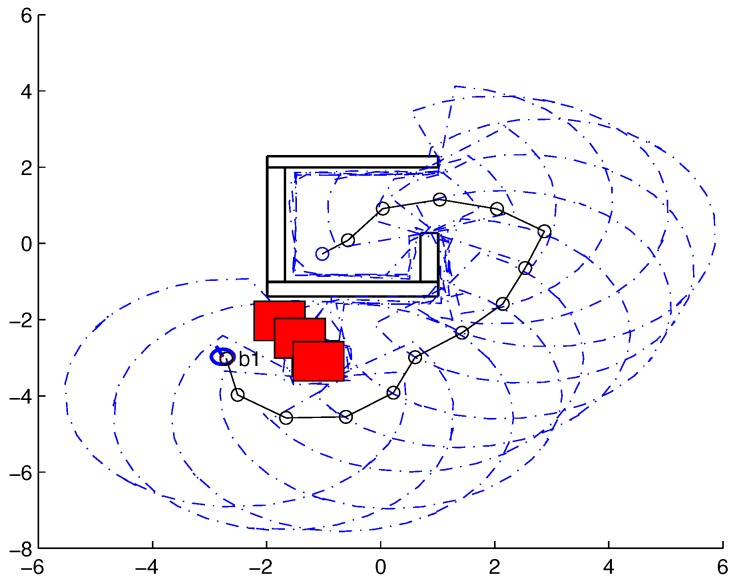
Displacement of the robot in simulation $S_{5}$ (J=30, H=10 e $R_{c}=80\%$). The red circumference—labeled as b1—indicates the final point of the displacement.

**Figure 10:**
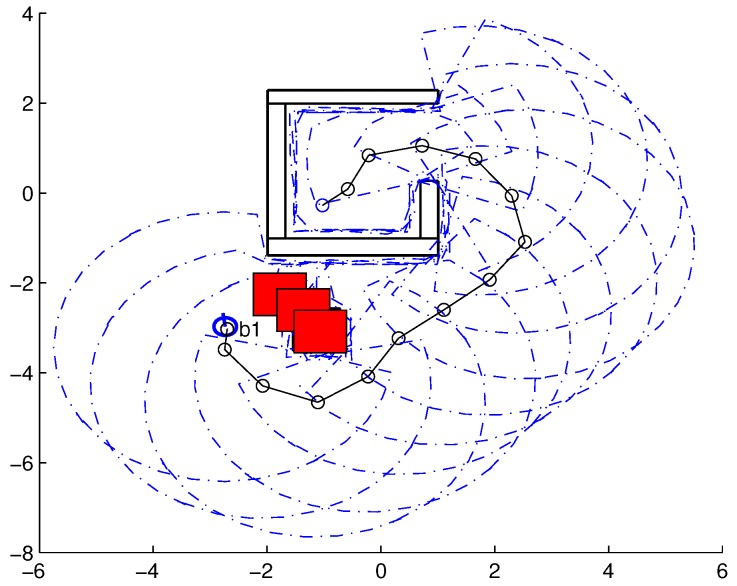
Displacement of the robot in simulation $S_{6}$ (J=10, H=30 e $R_{c}=80\%$). The red circumference—labeled as b1—indicates the final point of the displacement.

**Figure 11:**
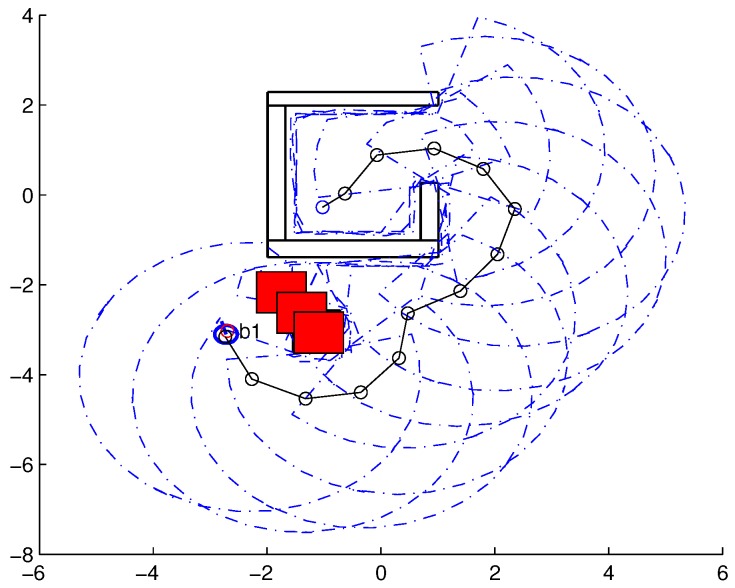
Displacement of the robot in simulation $S_{7}$ (J=30, H=10 e $R_{c}=60\%$). The red circumference—labeled as b1—indicates the final point of the displacement.

**Figure 12:**
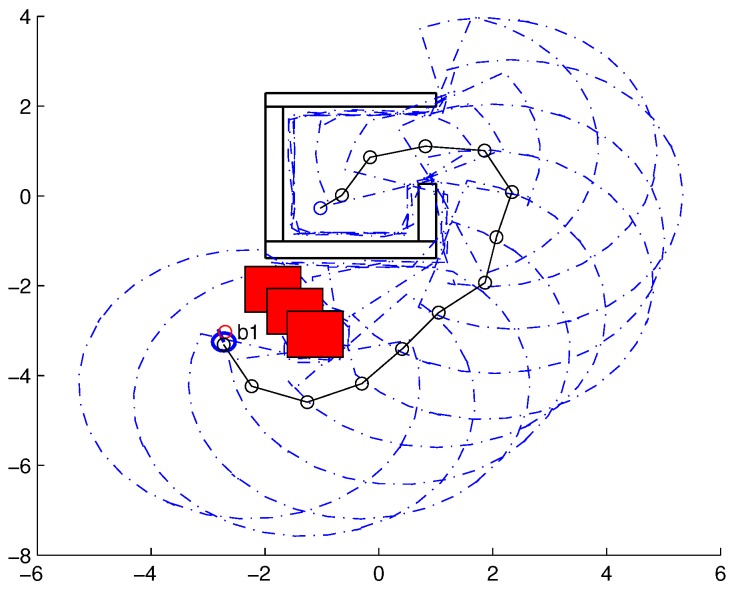
Displacement of the robot in simulation $S_{8}$ (J=10, H=30 e $R_{c}=60\%$). The red circumference—labeled as b1—indicates the final point of the displacement.

To validate the functioning of the DPNA-GA (for static and dynamic environments), considering its robustness in terms of the genetic parameters, simulations were conducted using two types of environment ($A_{1}$ and $A_{2}$), varying the number of generations (*H*), the size of the population (*J*), and the crossover rate ($R_{c}$). The simulations were performed in MATLAB, using the updated version of the iRobot Create toolbox [25,26]. The toolbox simulated a circular nonholonomic robot with variable action and four distance sensors spaced at $90^{\circ }$. Table 1 presents the fixed parameters used in the simulations. Each simulation (Figure 5, Figure 6, Figure 7, Figure 8, Figure 9, Figure 10, Figure 11 and Figure 12) was executed ten times, and the results are associated with the average of the values obtained in all executions.

The individuals used the real number encoding method, the crossover operator used the intermediate scheme where the offspring ($p_{i}^{GA}\left(h+1,m\right)$ and $p_{v}^{GA}\left(h+1,m\right)$) are chosen using the uniform random number, that is,

(20) $$p_{i}^{GA}\left(h+1,m\right)=p_{l}^{GA}\left(h,m\right)r\left(h,m\right)+p_{k}^{GA}\left(h,m\right)\left(1-r\left(h,m\right)\right)$$

and

(21) $$p_{v}^{GA}\left(h+1,m\right)=p_{l}^{GA}\left(h,m\right)\left(1-r\left(h,m\right)\right)+p_{k}^{GA}\left(h,m\right)r\left(h,m\right)$$

where the $p_{l}^{GA}\left(h,m\right)r_{j}\left(h,m\right)$ and $p_{k}^{GA}\left(h,m\right)r_{j}\left(h,m\right)$ are individuals chosen from $p^{GA}\left(h,m\right)$ in selection step and r(h,m) is a uniform random number between 0 and 1. Using Equation (9), Equations (20) and (21) can be rewritten as

(22) $$x_{i}^{GA}\left(h+1,m\right)=x_{l}^{GA}\left(h,m\right)r\left(h,m\right)+x_{k}^{GA}\left(h,m\right)\left(1-r\left(h,m\right)\right),$$

(23) $$x_{v}^{GA}\left(h+1,m\right)=x_{l}^{GA}\left(h,m\right)\left(1-r\left(h,m\right)\right)+x_{k}^{GA}\left(h,m\right)r\left(h,m\right),$$

(24) $$y_{i}^{GA}\left(h+1,m\right)=y_{l}^{GA}\left(h,m\right)r\left(h,m\right)+y_{k}^{GA}\left(h,m\right)\left(1-r\left(h,m\right)\right),$$

and,

(25) $$y_{v}^{GA}\left(h+1,m\right)=y_{l}^{GA}\left(h,m\right)\left(1-r\left(h,m\right)\right)+y_{k}^{GA}\left(h,m\right)r\left(h,m\right).$$

As the mutation operator, it was used the Gaussian mutation operator expressed as

(26) $$p_{i}^{GA}\left(h+1,m\right)=p_{i}^{GA}\left(h,m\right)+g\left(h,m\right)$$

where the g(h,m) is the Gaussian random variable of median zero and variance σ, N(0,σ), associated of the *h*-th generation of the *m*-th displacement of the robot. Using Equation (9), Equation (26) can be rewritten as

(27) $$x_{i}^{GA}\left(h+1,m\right)=x_{i}^{GA}\left(h,m\right)+g_{x}\left(h,m\right),$$

and,

(28) $$y_{i}^{GA}\left(h+1,m\right)=y_{i}^{GA}\left(h,m\right)+g_{y}\left(h,m\right),$$

where the $g_{x}\left(h,m\right)$ and $g_{y}\left(h,m\right)$ are the Gaussian random variable of median zero and variance σ, N(0,σ), associated of the *x* and *y* coordinates, respectively. In all simulations, it was used variance, σ=1.

Eight simulations were made, four for environment $A_{1}$ (simulations $S_{1}$, $S_{2}$, $S_{3}$, and $S_{4}$) and four for environment $A_{2}$ (simulations $S_{5}$, $S_{6}$, $S_{7}$, and $S_{8}$). For each simulation, Table 2 and Table 3 show the data for the length of the route, $c_{p}$, travelled by the robot (in meters), the processing time associated with all the displacements along the route, $t_{p}$ (in seconds), and the number of displacement events, *M*. The displacements of the robot in each simulation are illustrated in Figure 5, Figure 6, Figure 7, Figure 8, Figure 9, Figure 10, Figure 11 and Figure 12, where the route is indicated by a continuous black line, the *m* displacement events are shown as circles along the route lines, and the DPs associated with each displacement are indicated by dashed blue lines. The simulations were performed using a computer with a 64bits CPU (Intel(R) Core(TM) i5-3210M), 2.5GHz clock speed, and 8GBytes of RAM.

During the development of the work, several combinations of GA parameters were tested. How the target of the proposed method (DPNA-GA) is for embedded systems with low processing (such as microcontrollers), it was measured the time processing in seconds per displacement event (s/disp), $t_{d}$, for all simulations. After that, it was observed that the combinations with high population size (J>30), large generations number (H>30) and low crossover rate ($R_{c}<60\%$) achieved high values of td ($t_{d}>3\;s/\mathrm{disp}$). However, values of $t_{d}>3\;s/\mathrm{disp}$ can reduce the continuity of movement associated with the robot. Thus, the eight simulations ($S_{1}$ to $S_{8}$) were chosen using the criterion of $t_{d}<2.5\;s/\mathrm{disp}$ (time processing in seconds per displacement event).

The size of the population at J=10 is a relatively low amount for the GA standards, and increasing that amount generally implies a slight improvement in GA convergence values, but a considerable increase in processing time in environments with many obstacles. A similar situation is the one that concerns the crossover rate. Lowering the crossover rate, $R_{c}$, from 60% to close values or increasing the crossover rate, $R_{c}$, from 80% to close values, the results did not show significant differences. Lowering from 60% crossover rate to distant values has led to bad results that were already expected. Based on these results, we decided to include in our analysis only the most significant ones.

In the case of environment $A_{1}$ (results shown in Table 2 and Figure 5, Figure 6, Figure 7 and Figure 8), it can be seen that the navigation strategy showed little variation in terms of the number of displacement events, *m* (mean of 19.5 and standard deviation of 2.5), and the length of the route, $c_{p}$ (mean of 18.1m and standard deviation of 1.67m). However, greater variability was found for the processing time (mean of 28.92s and standard deviation of 12.88s).

Comparing simulations that have the same crossover rate (simulations $S_{1}$ and $S_{2}$, and simulations $S_{3}$ and $S_{4}$), it can be observed that the ones with a larger population size (simulations $S_{1}$ and $S_{3}$) have slightly higher performances in terms of route size and number of displacements. However, this little increase in performance does not compensate for the increase in processing time, which is more than twice its counterparts are. On the other hand, when comparing simulations with the same population size (simulations $S_{1}$ and $S_{3}$, and simulations $S_{2}$ and $S_{4}$), the increase in crossover rate meant a smaller route size and less displacements, with a less significant increase in processing time than in the previous comparison.

Lowering the crossover rate led to a more elitist configuration of the population, letting more individuals continue unchanged in the next generation. For a routing problem, reaching a few points in the search space can lead to a poor convergence of the algorithm. A larger population increases the variability of solutions reached in the search space, as can be seen in simulation $S_{3}$ (Figure 7), but it does not quite compensate for the lower crossover rate in this case. The simultaneous decrease of these parameters of genetic variability, as shown in simulation $S_{4}$ (Figure 8), leads to the worst convergence of the algorithm among the simulations, causing the robot to do bad and/or unnecessary displacements.

Finally, another important point to emphasize is that reduction of the size of the population only slightly increased the number of displacements (from 17 to 19) and greatly reduced the total time associated with the displacements (from 39.31s to 15.64s).

In the simulations using environment $A_{2}$ ($S_{5}$, $S_{6}$, $S_{7}$, and $S_{8}$), the robot encountered a dynamic obstacle (red rectangle) and had to avoid it. The results of these simulations are shown in Table 3 and Figure 9, Figure 10, Figure 11 and Figure 12. Different to the results for environment $A_{1}$, the data for the length of the route, $c_{p}$ (mean of 13.25m and standard deviation of 0.47m), the processing time, $t_{p}$ (mean of 10.43s and standard deviation of 0.67s), and the number of displacement events, *M* (mean of 13.5 and standard deviation of 0.58) showed very low variability associated with changes in the genetic parameters. This result could be explained by the alternation between the size of the population, *J*, and the number of generations, *H*, in simulations $S_{5}$, $S_{6}$, $S_{7}$, and $S_{8}$.

Compared to the findings for environment $A_{1}$, environment $A_{2}$ showed poorer results, with a crossover rate of 80%. This could be explained by the simplicity of environment $A_{2}$, relative to environment $A_{1}$, which did not require a high rate of renewal of the individuals of the population.

The data shown in Table 2 and Table 3 demonstrate that the execution time of the DPNA-GA is much shorter than for the strategies presented previously [6] for an environment similar to $A_{1}$. This difference is mainly associated with the size of each individual, the number of generations, and the size of the population, which in the case of the DPNA-GA were limited to 2, 30, and 30, respectively. The proposal described in [1], for example, employed populations of up to 2000 individuals with size of around 140 values (in the best case), for an environment similar to $A_{1}$. In other work, a population of 50 individuals was used, together with 2000 generations [11].

### Comparison with Other Approaches

**Figure 13:**
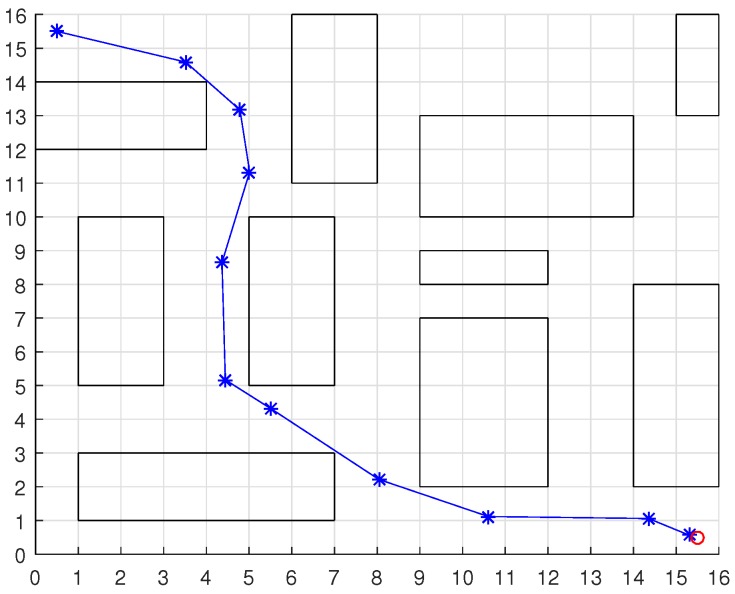
Displacement required for the robot using DPNA-GA in the environment proposed in Ref. [9]. The red circumference indicates the final point of the displacement.

**Figure 14:**
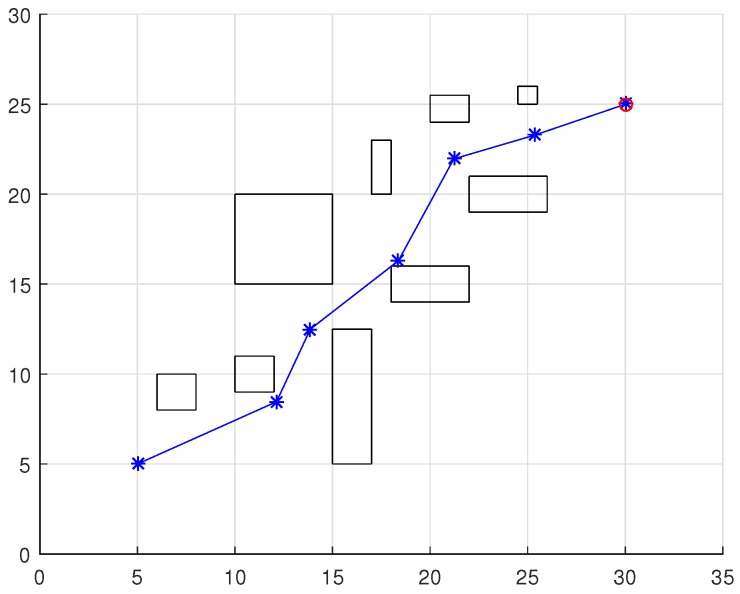
Displacement required for the robot using DPNA-GA in the environment proposed in Ref. [13]. The red circumference indicates the final point of the displacement.

**Figure 15:**
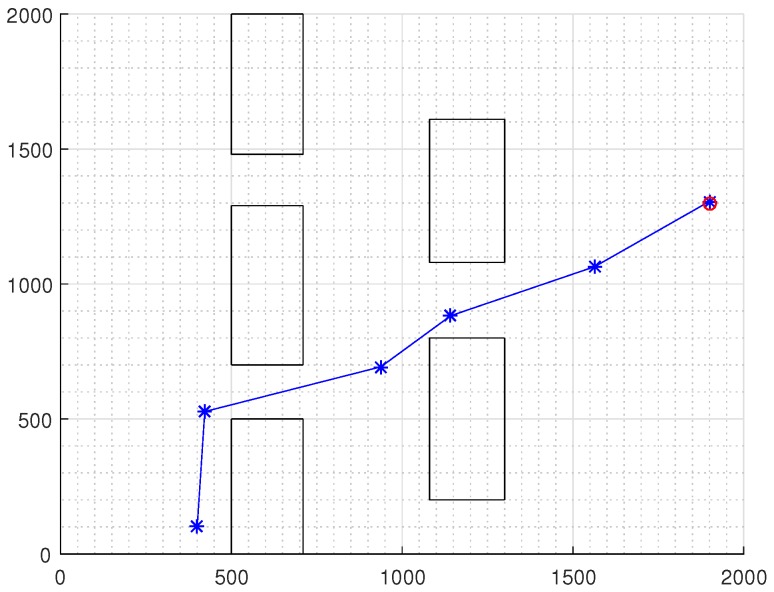
Displacement required for the robot using DPNA-GA in the environment proposed in Ref. [14]. The red circumference indicates the final point of the displacement.

**Figure 16:**
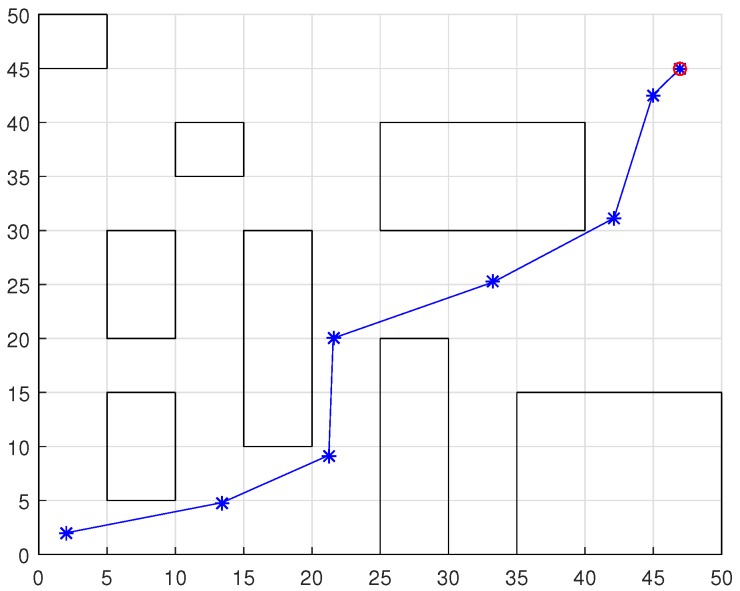
Displacement required for the robot using DPNA-GA in the environment proposed in Ref. [17]. The red circumference indicates the final point of the displacement.

To compare the results with other works in the literature, the DPNA-GA was simulated with environments used in works presented in Refs. [9,13,14,17,21]. Figure 13, Figure 14, Figure 15 and Figure 16 show the displacement of the DPNA-GA in the environment proposed in Refs. [9,13,14] and [17,21], respectively. Table 4 shows the parameters and the results of the DPNA-GA in the environments shown in the Figure 13, Figure 14, Figure 15 and Figure 16. Finally, Table 5 compares the result between the DPNA-GA and the literature works presented in Refs. [9,13,14,17]. Each simulation (Figure 13, Figure 14, Figure 15 and Figure 16) was executed ten times, and the results are associated with the best cases. The genetic parameters for DPNA-GA were J=30, H=10 and $R_{c}=80\%$, corresponding to the best configuration found in the simulations with static environment (see Table 2, $S_{2}$). The techniques compared were the Improved Genetic Algorithm (IGA) [9], the Matrix-Binary Codes-based Genetic Algorithm (MGA) [13], the Ant Colony Optimization (ACO) [14], the ACO with the Influence of Critical Obstacle (ACOIC) [14], the Firefly algorithm [17] and the Fuzzy System [21].

The comparative results in Table 5 show that the DPNA-GA had a route length gain in most cases. Table 6 presents the route length saved by DPNA-GA for works [13,14,17,21]. For research presented in Ref. [9] the DPNA-GA had a slightly worse result (<10%) however, it is important to emphasize that the work shown in Ref. [9] uses a GA navigation strategies with global planning in which each individual (or chromosome) is coded as a possible routes between the initial and final points and this increase the chromosome size and GA processing. The DPNA-GA uses the fixed chromosome size regardless of route length.

## 4. Conclusions

The objective of this work was to validate the robustness of a dynamic planning navigation technique for mobile terrestrial robots, based on genetic algorithms, denoted DPNA-GA. The validation was performed by varying some of the genetic parameters, in two different types of environment. Starting with strategies described in the literature as a basis, the DPNA-GA comprises a navigation scheme with local planning (applied to static and dynamic environments), in which the environment is unknown a priori and the size of the individuals is independent of the complexity of the environment. This property is fundamental from the point of view of practical implementation. The simulations showed that the DPNA-GA provided viable route solutions for different types of environment, following changes in the genetic parameters, hence demonstrating robustness at a relatively low cost, compared to other global and local planning strategies.

## Figures and Tables

**Table 1:** Common parameters used in the simulations.

Number of sensors (*n*)	4
Maximum sensor range ($d_{max}$) in meters	3 m
Angular displacement (α)	$10^{\circ }$
Radius ($r_{d}$) in meters	1 m
Z (Equation (17))	1000
Codification of individuals	Real number
Selection method	Stochastic uniform
Elitism	Yes (2 individuals)
Crossover operator	Uniform random [0,1]
Mutation operator	Gaussian random N(0,1)

**Table 2:** Parameters used in the simulations of environment $A_{1}$.

	Population Size (*J*)	Generations Number (*H*)	Crossover Rate (*R_c_*)	Length of Route (*c_p_*)	Processing Time (*t_p_*)	Displacement Events (*M*)	Time per Displacement (*t_d_*)
$S_{1}$	30	30	80%	16.52m	39.31s	17	2.31s/disp
$S_{2}$	10	30	80%	17.10m	15.64s	19	0.82s/disp
$S_{3}$	30	30	60%	18.46m	40.60s	19	2.14s/disp
$S_{4}$	10	30	60%	20.28m	20.15s	23	0.88s/disp

**Table 3:** Parameters used in the simulations of environment $A_{2}$.

	Population Size (*J*)	Generations Number (*H*)	Crossover Rate (*R_c_*)	Length of Route (*c_p_*)	Processing Time (*t_p_*)	Displacement Events (*M*)	Time per Displacement (*t_d_*)
$S_{5}$	30	10	80%	13.94m	10.65s	14	0.76s/disp
$S_{6}$	10	30	80%	13.19m	11.25s	14	0.80s/disp
$S_{7}$	30	10	60%	12.87m	9.68s	13	0.74s/disp
$S_{8}$	10	30	60%	12.93m	10.16s	13	0.78s/disp

**Table 4:** Results of the simulations of environments shown in the Figure 13, Figure 14, Figure 15 and Figure 16.

Environment		Length of Route (*c_p_*)	Processing Time (*t_p_*)	Displacement Events (*M*)	Time per Displacement (*t_d_*)
Reference [9]	Figure 13	25.95	15.83s	10	1.58s/disp
Reference [13]	Figure 14	33.92	7.92s	6	1.32s/disp
Reference [14]	Figure 15	2119.94	8.39s	5	1.68s/disp
Reference [17,21]	Figure 16	69.93	10.51s	7	1.50s/disp

**Table 5:** Route length comparison between DPNA-GA and other navigation approaches.

Environment		Technique	Other Approaches		DPNA-GA	
			Length of the Best Route	Average Route Length	Length of the Best Route	Average Route Length
Reference [9]	Figure 13	IGA	22.83	22.83	25.05	25.95
Reference [13]	Figure 14	MGA	35.1	35.1	33.92	36.77
Reference [14]	Figure 15	ACO	2700	3513.3	2119.94	2720.74
		ACOIC		3446.7		
Reference [17]	Figure 16	Firefly	95	95	69.93	72.96
Reference [21]		Fuzzy	97.97	98.53		

**Table 6:** The route length, in %, saved by DPNA-GA.

Environment		Technique	Route Length Saved by DPNA-GA
Reference [13]	Figure 14	MGA	3.47%
Reference [14]	Figure 15	ACO	27.36%
		ACOIC	
Reference [17]	Figure 16	Firefly	37.28%
Reference [21]		Fuzzy	40.00%
